# Rational improvement of gp41-targeting HIV-1 fusion inhibitors: an innovatively designed Ile-Asp-Leu tail with alternative conformations

**DOI:** 10.1038/srep31983

**Published:** 2016-09-26

**Authors:** Yun Zhu, Shan Su, Lili Qin, Qian Wang, Lei Shi, Zhenxuan Ma, Jianchao Tang, Shibo Jiang, Lu Lu, Sheng Ye, Rongguang Zhang

**Affiliations:** 1 National Laboratory of Biomacromolecules, Institute of Biophysics, Chinese Academy of Sciences, Beijing, 100101, China; 2 Key Laboratory of Medical Molecular Virology of Ministries of Education and Health, Basic Medical College and Shanghai Public Health Clinical Center, Fudan University, Shanghai, 200032, China; 3 Lindsley F. Kimball Research Institute, New York Blood Center, New York, New York 10065, USA; 4 National Center for Protein Science Shanghai, Institute of Biochemistry and Cell Biology, Shanghai Institutes for Biological Sciences, Chinese Academy of Sciences, Shanghai, 201210, China

## Abstract

Peptides derived from the C-terminal heptad repeat (CHR) of HIV gp41 have been developed as effective fusion inhibitors against HIV-1, but facing the challenges of enhancing potency and stability. Here, we report a rationally designed novel HIV-1 fusion inhibitor derived from CHR-derived peptide (Trp628~Gln653, named CP), but with an innovative Ile-Asp-Leu tail (IDL) that dramatically increased the inhibitory activity by up to 100 folds. We also determined the crystal structures of artificial fusion peptides N36- and N43-L6-CP-IDL. Although the overall structures of both fusion peptides share the canonical six-helix bundle (6-HB) configuration, their IDL tails adopt two different conformations: a one-turn helix with the N36, and a hook-like structure with the longer N43. Structural comparison showed that the hook-like IDL tail possesses a larger interaction interface with NHR than the helical one. Further molecular dynamics simulations of the two 6-HBs and isolated CP-IDL peptides suggested that hook-like form of IDL tail can be stabilized by its binding to NHR trimer. Therefore, CP-IDL has potential for further development as a new HIV fusion inhibitor, and this strategy could be widely used in developing artificial fusion inhibitors against HIV and other enveloped viruses.

HIV entry into a host cell requires a critical membrane fusion process mediated by HIV envelope glycoprotein (Env) transmembrane subunit gp41^1^,^2^, a direct target of HIV fusion inhibitors that are usually adopted to treat the AIDS patients without response to other antiretroviral therapeutics^3^,^4^,^5^. Most of HIV fusion inhibitors against gp41 are peptides derived from the helical C-terminal heptad repeat (CHR) of gp41, including a number of currently experimental drugs and T20 (Enfuvirtide, Fuzeon), the first and only gp41-targeting anti-HIV agent approved by the U.S. FDA. These CHR-like peptidic inhibitors in helical form are capable to specifically bind to the homotrimeric gp41 N-terminal heptad repeat (NHR), interfering the formation of a NHR-CHR six-helix bundle (6-HB), which has been proved to be the key step leading to the virus and cell membrane fusion^6^,^7^,^8^. Structural studies have confirmed that both the assembly of natural HIV 6-HB and the binding affinity between fusion inhibitors and gp41 NHR predominantly rely on inter-helix hydrophobic interactions^9^,^10^.

Unfortunately, the clinical application of T20, as well as other similar HIV fusion inhibitors, has been severely limited by, among others, two major factors: its low potency and high susceptibility to drug resistance^9^,^10^,^11^. To conquer these problems, tremendous efforts have been made to optimize the CHR-derived inhibitor helices, including, but not limited to, i) introducing more salt bridges at the outer side to improve their stability^5^; and ii) substitution of hydrophobic residues at the inner side to enhance their affinity with the hydrophobic groove between two NHR helices^12^,^13^,^14^. In addition to the modification of the inhibitor helix itself, the existence of a native N-terminal hook-like structure formed by Met626 and Thr627 (MT hook) have been evidenced to enhance the antiviral activity of CHR-derived inhibitors by approximately 10-fold^15^,^16^. Moreover, structural studies revealed that the hydrophobic side chain of the methionine residue in MT hook participates in hydrophobic interactions with both the NHR trimer and the CHR-derived inhibitor.

Inspired by the gp41-derived MT hook, we aimed to append a rationally designed artificial tail to the C-terminus of HIV-1 fusion inhibitors to enhance their antiviral potency. Finally, by introducing an innovative C-terminal tail of Ile-Asp-Leu (IDL), we succeeded to increase the anti-HIV potency of a CHR-derived peptide (Trp628~Gln653, named CP, Fig. 1A) by more than 100-fold. Unexpectedly, the crystal structures of CP-IDL in complex with NHR helices at different lengths (N36: Ser546~Leu581, and N43: Val539~Leu581, Fig. 1A) show that IDL tail is capable to bear two different conformations: a part of an α-helix (with N36) or a hook-like structure (with N43). Further structural analysis and molecular dynamic (MD) simulations suggested that the alternative conformations of IDL tail allow the enhancement of the anti-HIV potency of CP-IDL peptide. Based on the present study, we believe that similar approaches are possible to be adopted in the improvement of CHR-derived fusion inhibitors against other viruses.

## Results

### N-terminal hydrophobic pocket of gp41 NHR

It is known that, in an HIV-1 6-HB, each CHR helix is half buried in the hydrophobic groove between two adjacent helices of NHR homotrimer, assembling the so-called fusion core. After careful examination of previously reported crystal structures, we noticed that, outside the NHR hydrophobic groove, a solvent exposed hydrophobic pocket is located around the N-terminus of NHR (close to the binding site of CHR Gln653, Fig. S1A). Since this N-terminal hydrophobic pocket (NTHP) does not lie in the hydrophobic groove, it cannot be covered by canonical CHRs or CHR-derived peptides (Fig. S1A). The NTHP is majorly composed of four hydrophobic residues, including Leu544 and Ile548 from one NHR helix, and Leu545 and Val549 from an adjacent NHR helix (Fig. S1B). Amino acid sequence alignment showed that those residues are highly conserved among different HIV-1 strains (Fig. S1C), implying that this previously undescribed pocket could be a potential target for enhancing the inhibitory potency of CP peptide.

### CP-IDL peptide has improved anti-HIV-1 potency

**Figure 1:**
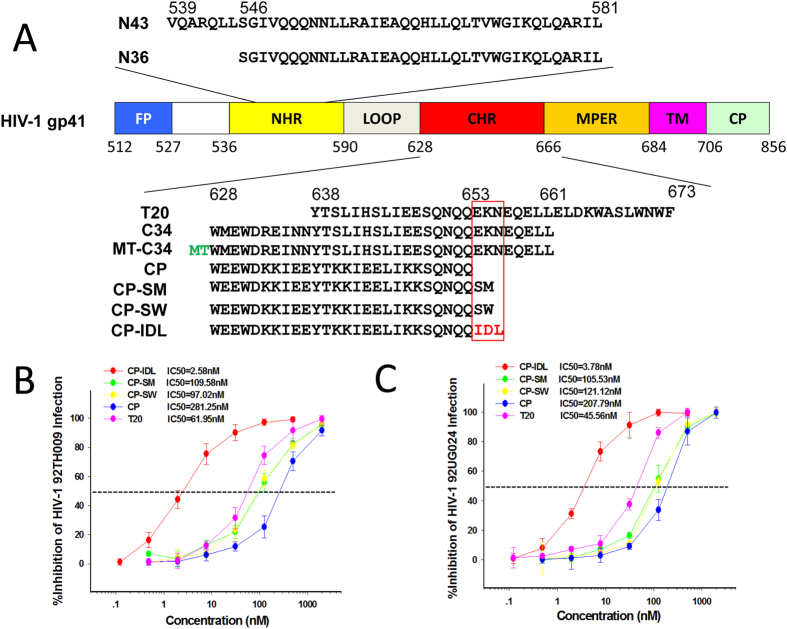
HIV-1 gp41 CHR-derived peptides and their inhibition activities. **(A**) Schematic illustration of HIV-1 gp41 functional regions and NHR- or CHR-derived peptide sequences. The residue numbers of each region correspond to their positions in gp160 of HIV-1HXB2. FP, fusion peptide; CP, cytoplasmic peptide. The MT hook residues in the N terminus of CHR are marked in green. The IDL hook residues in the C terminus are marked in red. (**B**) Inhibition activities of CP-IDL, CP-SM, CP-SW, CP and T-20 to HIV-1 clinical strain 92UG029 infecting M7 cells. (**C**) Inhibition of CP-IDL, CP-SM, CP-SW, CP and T-20 to HIV-1 clinical strain 92UG024 infecting M7 cells.

To create a C-terminal tail possessing interactions with N-terminal hydrophobic pocket (NTHP), we truncated the CHR-derived peptide as Trp628 ~ Gln653, resulting in CP (Fig. 1A). Then a number of two- or three-residue tails incorporating hydrophobic side chains were rationally designed to fit the shape of NTHP, including Ile-Asp-Leu, Ser-Met and Ser-Trp, leading to peptidic inhibitors CP-IDL, CP-SM and CP-SW, respectively. Subsequently, the four peptides (with or without designed tails), together with T20, were artificially synthesized and subjected to the assessment of their antiviral activities (Fig. 1B,C). The original CP peptide has low inhibitory activities against HIV-1 clinical strains, 92TH009 (subtype A/E, R5) and 92UG024 (subtype D, X4), with IC_50_ of 281.25 nM and 207.79 nM, respectively. As expected, at the presence of artificial C-terminal tails, the inhibitory activities of elongated CPs were raised to different extends, where CP-IDL showed the highest potency with increase of 109- and 55-fold for 92TH009 and 92UG024, respectively. Moreover, the enhanced activities of CP-IDL were stronger than that of T20 by 24- and 12-fold for the two strains. Then we compared the inhibitory activities of CP, CP-IDL and T20 against more HIV-1 isolates, including tire1, 2 and 3 viruses (Table 1). It showed that CP-IDL could inhibit infection of all those different types of HIV-1 pseudovirus with IC_50_ values between 1.21 nM and 74.49 nM, which were much more potent than CP peptide (3.1 fold to 52 fold) or T20 (2.8 fold to 21.6 fold). Then we tested the inhibitory activities of CP, CP-IDL and T20 against T2635 or T20 resistant strains (Table 2). It showed that CP-IDL could inhibit infection of all these resistant strains, which are much more potent than CP or T20. The fact that the anti-HIV-1 potency of CP inhibitor could be remarkably improved by a C-terminal IDL tail implied that this three-residue tail binds to NHR in a novel interaction pattern other than the classical helix-groove one.

### Alternative conformations of CP-IDL peptide

**Figure 2:**
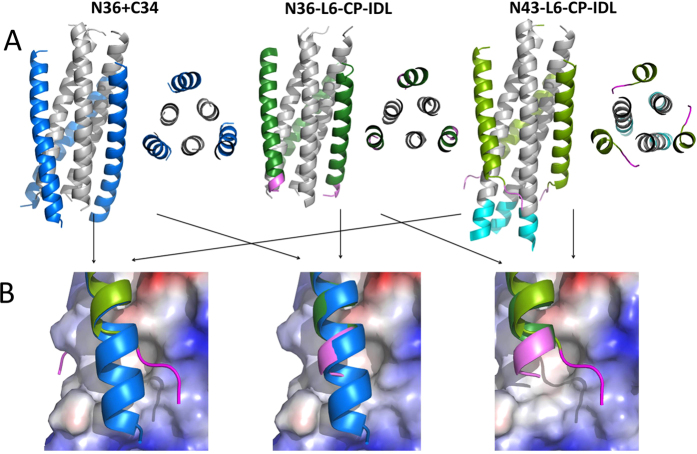
Two different conformations of IDL tail in fusion inhibitor CP-IDL. (**A**) Crystal structure of N36-L6-C34 (PDB: 1AIK), N36-L6-CP-IDL and N43-L6-CP-IDL, shown in both side view and cross-section view. NHR is colored in grey, C34 in blue. In N36-L6-CP-IDL, CP in forest and IDL in violet. In N43-L6-CP-IDL, CP in split pea and IDL in magentas, extend region of N43 in cyan. (**B**) Superposition of two crystal structures in the tail region, NHR are shown as electrostatic surface.

**Figure 3:**
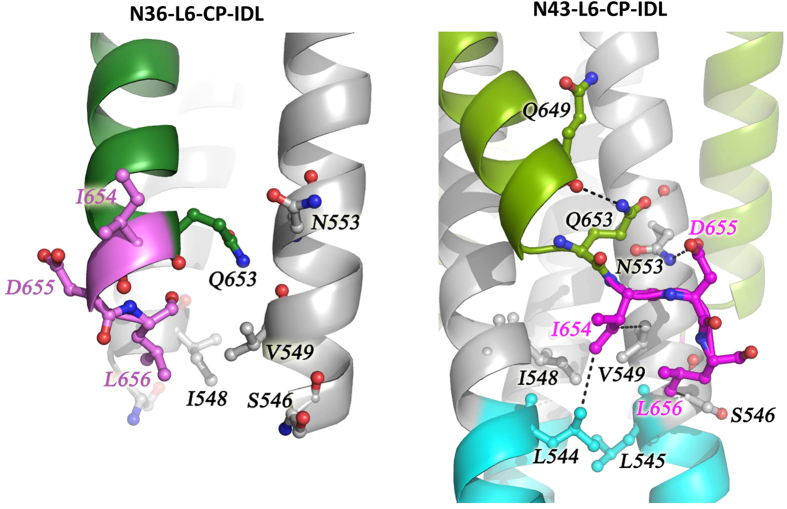
Residue interactions in the tail region of CP-IDL in two conformations. Crystal structure of N36-L6-CP-IDL and N43-L6-CP-IDL are shown as cartoon representation. NHR is colored in grey, In N36-L6-CP-IDL, CP in forest and IDL in violet. In N43-L6-CP-IDL, CP in split pea and IDL in magentas, extend region of N43 in cyan. Important residues are shown in sticks and labeled.

To explore the exact binding mode of the artificial IDL tail, we first determined the crystal structure of a fusion peptide N36-L6-CP-IDL, where N36 is a NHR peptide widely employed in the structural studies of HIV-1 fusion core. Unexpectedly, well-defined electron density (Fig. S2A) shows that the whole CP-IDL folds into a continuous α-helix (Fig. 2A), which can be perfectly superimposed to the C34 peptide in 6-HB structure (Fig. 2B), despite the significant difference between their tail sequences (the counterpart of Ile-Asp-Leu in C34 is Glu-Lys-Asn, Fig. 1A). By forming almost one turn of the helix, the isoleucine and aspartic acid residues of IDL are located at the outer side and hence have no interactions with N36, while the leucine residue participates in hydrophobic interactions with Ile548 and Val549 from two adjacent NHR helices, respectively (Fig. 3A). The interface area between one CP-IDL and trimeric NHR is 1098~1218 Å^2^, where IDL tails contribute 66~124 Å^2^ (approximately 10%). In addition, we noticed that, “below” the N-terminus of N36, there are plenty of space allowing more residues from NHR to interact with IDL tail, of which the structural features cannot be unveiled by the structure of N36-L6-CP-IDL.

IDL was designed to target N-terminal hydrophobic pocket (NTHP), which is mainly composed of Leu544, Leu545, Ile548 and Val549. But N36 (Ser546~Leu581) trimer does not contain the intact NTHP target. Therefore, we further determined the crystal structure of N43-L6-CP-IDL, where N43 (Val539~Leu581) is 7-residue longer at N-terminus than N36, and contains whole target of NTHP (Fig. 1A). In the final model, although the CP portion still folds into an α-helix, the IDL tail adopts a totally different conformation, forming a hook-like structure packing against the N-terminal hydrophobic pocket (NTHP) (Fig. 2A), just as we designed it to. The side chain orientations of both the isoleucine and leucine residues in IDL tail are toward the NHR trimer, possessing hydrophobic interactions with conserved hydrophobic residues in NTHP, including Leu545 and Val549 from the NHR helix within the same fusion peptide, and Leu544 and Ile548 from an adjacent NHR helix. The hydrophilic aspartic acid residue in IDL tail also forms an interaction with NHR by contributing its side chain oxygen atom to form a salt bridge (3.2 Å) with the side chain nitrogen atom of Asn553 (Fig. 3B). Besides the three tail residues themselves, their conformational changes also influence the side chain orientation of Gln653, the very residue prior to the IDL tail, resulting in a hydrogen bond (3.1 Å) with the main chain oxygen atom of Gln649, further stabilizing the hook-like IDL tail. As a consequence, in the structure of N43-L6-CP-IDL, binding of one CP-IDL leads to a buried surface area of 1115~1159 Å^2^, where IDL hook-like tail contributes 158~165 Å^2^ (approximately 13.9~14.4%), larger than the contribution of helical IDL tails in the structure of N36-L6-CP-IDL. Compared to the helical form, the hook-like IDL exhibited more extensive interactions with NHR and, hence, is more likely to represent the natural conformation when CP-IDL binds to homotrimeric NHR.

It is worth noting that both the structures of N36- and N43-L6-CP-IDL contain 6 chains of fusion peptides within one asymmetric unit, forming two 6-HBs. For each structure, the 6 chains are generally identical (r.m.s. deviations of 0.38~0.88 Å and 0.12~0.30 Å for N36- and N43-L6-CP-IDL, respectively, Table S1), but with conformational differences that cannot be ignored. When solving the structure of N43-L6-CP-IDL, the space group was assigned as P6_3_ at first, but resulting in unacceptable refinement statistics (R_free_ > 0.30). Then, reassigning the space group as P1 solved the problem. In the final model, six IDL tails in one asymmetric unit adopted similar hook-like conformations with different B-factors (Table S2), indicating its flexibility when binding to NHR trimer. As for N36-L6-CP-IDL, even one of the six helical IDL tails is missing in the final model. Considering that, in both structures, all of the 6HBs are compactly packed against one another, leading to direct interactions between most IDL tails and their adjacent 6HBs (Fig. S3A,B), it is necessary to investigate whether the IDL structures we observed are artificial states resulted from crystal packing.

### Molecular dynamics simulations of CP-IDL peptide

**Figure 4:**
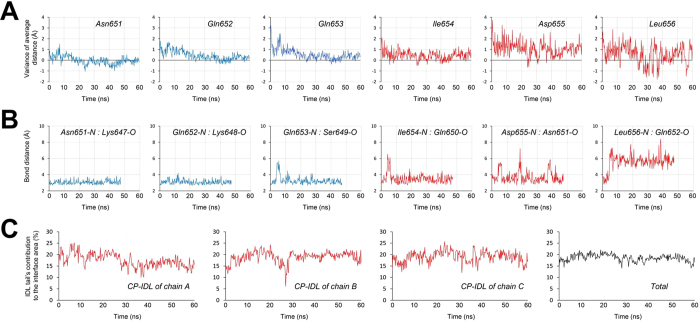
Molecular Dynamic simulations of 6-HBs. **(A**) Variance of the average Cα-Cα distance between a C-terminal residue of CP-IDL and all residues of an adjacent NHR helix during one simulation run of 60 ns performed on an N43-L6-CP-IDL 6HB model. (**B**) Lengths of the hydrogen bonds responsible for the α-helical conformation of the IDL tails in a N36-L6-CP-IDL 6HB model. (**C**) Contributions of individual IDL tails to the buried surface area between CP-IDL and NHR trimer in an N43-L6-CP-IDL 6HB model during one simulation run of 60 ns, as well as their sum. See methods for details.

To assess the stabilities of two IDL conformations, molecular dynamics (MD) simulations were performed on two 6-HB models from the crystal structures of N36- and N43-L6-CP-IDL, respectively, as well as isolated CP-IDL peptides in the two structures. In 2 runs of 60 ns we conducted, the hook-like structure of the IDL tails was generally retained in an N43-L6-CP-IDL 6HB. The positions of 6 C-terminal residues were assessed by calculating the variance of their distances to the NHR within the same fusion peptide (average Cα-Cα distance). As shown in Fig. 4A, residue Ile654 of IDL tail was as tightly fixed as the residues prior to it, while the other two residues of IDL tail, Asp655 and Leu656, were more flexible. However, the interface area between a hook-like IDL tail and an NHR trimer was roughly stable in the range of 150~300 Å^2^, contributing 12~25% of the interface between CP-IDL and NHR (Fig. 4B). Interestingly, the total interface area of three IDL tails in the same 6-HB is more stable than individual tails, possessing a contribution of 15~22% (Fig. 4B). As for the IDL tails in an N36-L6-CP-IDL 6HB, analysis of the hydrogen-bonds responsible for the α-helical conformation of the C-terminus showed that the helical conformation could be stabilized during 2 runs of 115 ns (Fig. 4C). However, without the presence of NHR trimer, neither helical nor hook-like structure of the IDL tail could be maintained for more than 10 ns in the 4 runs we performed on each peptide (Movie S1). In conclusion, MD simulations indicated that the IDL tail in a separated CP-IDL peptide tend to bear a disordered conformation, which will be altered to a stable helical or hook-like structure when binding to short or long NHR helices, respectively.

## Discussion

The 6HB fusion core is a critical target of antivirus fusion inhibitors, most of which are peptides derived from CHR and, hence, are in helical conformations. Many approaches have been proven to effectively improve those inhibitors, including mutant screening, chemical modifications, conjugation with proteins, and etc.^17^,^18^,^19^, none of which, however, was rationally designed to alter the helical conformation of the original inhibitors or to target a novel binding site other than the hydrophobic grooves between two adjacent NHR helices. Recently, multiple studies showed that motifs with non-helical structures were also capable to improve the potency of helical fusion inhibitors and even could be more crucial than the helix itself. For anti-HIV CHR-derived peptides, a hook-like structure formed by two native N-terminal CHR residues, Met626 and Thr627, could increase the antiviral activity of both modified and unmodified inhibitors^15^,^20^. In a previously published work relating to the Middle East respiratory syndrome coronavirus, based on the crystal structure of its 6HB fusion core, we successfully designed an effective inhibitory peptide HR2P, of which two non-helical tails at both ends are extremely necessary to its antiviral potency^21^. In the present work, we are showing a novel case of rationally designed non-helical C-terminal IDL tail, which is capable to effectively enhance the potency of anti-HIV-1 CHR-derived inhibitors by forming a hook-like structure tightly packed against the NTHP, a novel binding site close to the canonical hydrophobic grooves. These recent progress suggest that adding non-helical tails at the N- or C-terminus might be a valid and universal strategy to improve the antiviral potency of fusion inhibitors.

For the IDL tail in anti-HIV-1 fusion inhibitors, we crystallographically observed two distinguished conformations, a one-turn helix and a hook-like structure. Given that the IDL hook was crystallized with a longer NHR peptides and contributes larger interface area than the helical one does, we believed that the structure with hook-like IDL tail is more likely to represent the natural conformation of CP-IDL:NHR complexes in solution. However, according to our MD simulation results, both the helical and hook-like structures of IDL tail can be stabilized by their interactions with an NHR trimer. Considering that the shifts of residues Asp655 and Leu656 between two conformations are 9.2 and 8.5 Å, respectively (Fig. S4), the conformational transition between the two states seems impossible to spontaneously occur in an assembled 6-HB. Previous studies have confirmed that an isolated CP peptide shows low α-helicity, indicating that its both ends are relatively disordered in solution^22^, in agreement with our MD simulations of isolated CP-IDL peptides. Therefore, we propose that, before combining with homotrimeric NHRs, the IDL tail of free CP-IDL peptide is generally disordered, which will be altered to a relatively ordered hook-like structure by its binding to an NHR trimer, since the hook-like tail is conformationally similar to the previously disordered tail.

It has been more than a decade since T20, the first and only HIV fusion inhibitor targeting gp41, was proved by U.S. FDA as an anti-HIV agent in the combination therapy of AIDS^23^,^24^. However, in recent years, its shortcomings have limited its clinical application, including the lower anti-HIV activity, shorter half-life, expensive synthetic cost, and emerged T20-resistant HIV-1 variants^25^. So, it is necessary to design new upgraded inhibitors, with better inhibitory activities, or with shorter length to reduce the synthetic costs, or with artificial sequences different from CHR, or with enlarged binding targets. As a result of such kind of rational design, we obtained the fusion inhibitors with a hook-like IDL tail, an innovative solution to the problems of T20 and similar inhibitors. In addition, compared to T20 (36 a.a.), CP-IDL has a shorter length of 29 a.a., possessing a notably reduced synthetic cost. With no doubt, CP-IDL is a potential candidate for further development as a new anti-HIV fusion inhibitor for clinical applications. Moreover, our results highlighted the advantages of artificially designed peptides, which could be widely employed in the development of artificial peptide-based virus fusion inhibitors against HIV-1 and other enveloped viruses with class I membrane fusion proteins, such as SARS-CoV^26^, MERS-CoV^21^, and paramyxovirus^27^.

## Methods

### Peptide synthesis

A panel of peptides (Fig. 1A), including T20, CP, CP-SM, CP-SW, CP-IDL were synthesized with a standard solid-phase FMOC method, as described previously^5^. The peptides were found to be about 95% pure by HPLC and were identified by mass spectrometry (Perseptive Biosystems, Framingham, MA, USA). Concentrations of the peptides were determined by UV absorbance and a theoretically calculated molar-extinction coefficient based on tryptophan and tyrosine residues.

### Inhibition of peptides on HIV-1 clinical strains

Inhibition activities of peptides on infection by HIV-1 clinical strains were determined as previously described^28^. For inhibition of HIV-1 92TH009 (subtype A/E, R5) and 92UG024 (subtype D, X4) infection,100 TCID_50_ of the virus was added to 1 × 10^5^/ml M7 cells in RPMI 1640 medium containing 10% FBS in the presence or absence of the test peptide overnight. Then, the culture supernatants were changed to fresh media. On the fourth day post-infection, culture supernatants were collected for detection of p24 antigen by ELISA. The percent inhibition of p24 production was calculated using the computer program Calcusyn.

### Expression and purification of fusion protein

Using overlapping PCR, the DNA fragment encoding CP-IDL peptide was attached to the 3′-end of the cDNA of gp41 NHR (N36, 546–581 or N43 539–581), with a short linker (L6, SGGRGG) between them. Then, the whole sequence was subcloned into the pET-32p vector (Novagen, USA). The pET-32p-N36-L6-CP-IDL- or pET-32p-N43-L6-CP-IDL-transformed *E. coli* cells were induced by adding 1 mM IPTG and incubating overnight at 16 °C. Fusion protein was purified by Ni-NTA affinity resin (Qiagen, Valencia, CA, USA), and the Trx-tag was cleaved off by Prescission Protease enzyme treatment at 4 °C for 2 h. The purified N36-L6-CP-IDL or N43-L6-CP-IDL was applied onto a Superdex-75 gel filtration column (GE Healthcare, Piscataway, NJ, USA). Fractions containing N36-L6-CP-IDL or N43-L6-CP-IDL trimer were collected and concentrated to different concentrations by ultrafiltration.

### Crystallization, data collection, and structure determination

The fusion protein N36-L6-CP-IDL was crystallized at 16 °C using the hanging drop, vapor-diffusion method. The drops were set on a siliconized cover clip by equilibrating a mixture containing 1 μl protein solution (25 mg/ml N36-L6-CP-IDL trimer in 20 mM Tris-HCl pH 8.0 and 150 mM NaCl) and 1 μl reservoir solution (0.2 M NaCl, 0.1 M Na_2_HPO_4_:citric acid, pH 4.2, 15–20% (w/v) PEG 3000) against a 400 μl reservoir solution. After one week, single crystals formed and were flash frozen by liquid nitrogen for future data collection. Fusion protein N43-L6-CP-IDL was crystallized in a similar way with a different reservoir solution (1.4 M Sodium Potassium Phosphate pH 8.2). After two weeks, single crystals formed and were flash frozen by liquid nitrogen for future data collection.

The datasets of N36-L6-CP-IDL and N43-L6-CP-IDL were both collected on an in-house x-ray source (MicroMax 007 x-ray generator, Rigaku, Japan) at the Institute of Biophysics, Chinese Academy of Sciences. X-ray diffraction data were integrated and scaled using the HKL2000 program^29^. The phasing problem of all three structures was solved by the molecular replacement method using PHENIX.phaser^30^ with the crystal structure of HIV gp41 NHR-CHR (PDB entry: 1SZT) as a search model. The final models were manually adjusted in COOT^31^ and refined with PHENIX.refine^32^. The space group of N43-L6-CP-IDL was first assigned as P6_3_ with one peptide in one asymmetric unit, which, however, leaded to unacceptable statistics (R_free_ > 0.30 at 2.3 Å). The problem was then solved by reassigning the space group as P1 with six chains in one asymmetric unit. All coordinates were deposited in the Protein Data Bank (N36-L6-CP-IDL: 5HFL; N43-L6-CP-IDL: 5HFM). The statistics of data collection and structure refinement are given in Table 3.

### Molecular Dynamic simulations

Preliminary MD simulations for the modeled protein were performed using the program NAMD (NAnoscale Molecular Dynamics program; v 2.9)^33^ and CHARMM22 force field^34^, and all files were generated using visual molecular dynamics (VMD)^35^. The 6-HBs containing chains A, B and C of both N36- and N43-L6-CP-IDL structures were chosen to be the initial models. Hydrogen atom coordinates of both models were generated by the AutoPSF plugin of VMD. Then the protein models were solvated by a TIP3P water box with a 20-Å layer of water molecules at each direction and then ionized with sodium and chloride ions to the physiological concentration of 0.15 M. Each model was subjected to energy minimization of 50,000 steps, followed by restrained NPT equilibration of 5 ns with isobaric and isothermal conditions maintained at 1 atm and 350 K. Subsequently, production simulations were conducted on equilibrated models for proper duration (60~115 ns) with an integration time step of 2 fs. The structure of N36-L6-CP-IDL and isolated CP-IDL peptide models were subjected to MD simulations in the same fashion as described above. All simulation runs were performed on the ScGrid of the Supercomputing Environment of the Chinese Academy of Sciences.

### Simulation analysis

Visualization and extraction of raw trajectory data for analysis were performed using VMD. To assess the stability of the interactions between IDL tail and its combined NHR trimer, the average Cα-Cα distance between each C-terminal residue of CP-IDL and all NHR residues was calculated. Then, the variance of the distance during simulation runs was monitored and plotted. In addition, the contribution of IDL tails to the buried areas were also calculated and monitored during simulation runs, where the buried area was simply considered as one half of the solvent accessible surface area difference between complex A:B and the sum of A and B. When calculating the solvent accessible surface, a typical probe radius of 1.4 Å was employed. Two Tcl scripts were coded and performed in VMD Tk console to realize the calculations described above.

## Additional Information

**How to cite this article**: Zhu, Y. *et al*. Rational improvement of gp41-targeting HIV-1 fusion inhibitors: an innovatively designed Ile-Asp-Leu tail with alternative conformations. *Sci. Rep.* **6**, 31983; doi: 10.1038/srep31983 (2016).

## Supplementary Material

Supplementary Information

Supplementary Movie 1

## Figures and Tables

**Table 1:** Inhibitory activities of CP, CP-IDL and T20 against HIV-1 pseudovirus.

Virus	Subtype	Coreceptor Usage	Tier	IC50 (nM) of		
				CP	CP-IDL	T20
SF162.LS	B	R5	Tier 1	78.79 ± 11	1.51 ± 0.4	25.56 ± 3.1
CRF02_AG clone 242	AG	R5	Tier 1	61.16 ± 13	6.24 ± 3.7	17.63 ± 2.7
pCH119 env	BC	R5	Tier 2	42.29 ± 2.9	5.79 ± 1.1	28.79 ± 2.4
CRF02_AG/A1 clone 280	AG/A1	R5	Tier 2	213.3 ± 14	5.62 ± 1.2	28.39 ± 3.2
CRF02_AG clone 278	AG	R5	Tier 3	44.39 ± 13	8.59 ± 2.4	78.79 ± 11
CRF02_AG clone 33	AG	R5	Tier 3	111.2 ± 4.8	2.82 ± 0.5	45.73 ± 1.6
BG505.W6M.ENV.C2	A	N	N	26.46 ± 8.6	8.61 ± 2.8	33.24 ± 8.5
B1206.W6P.ENV.A1	A	N	N	>2000	74.49 ± 12	517.6 ± 23
BJ412.W6M.ENV.S3	C	N	N	51.86 ± 12	1.21 ± 0.1	26.17 ± 5.4
ZM109F.PB4	C	R5	N	63.39 ± 4.6	2.93 ± 1.2	19.35 ± 4.4
MJ412.W0M.ENV.B1	C	N	N	45.98 ± 3.2	2.24 ± 1.3	29.23 ± 4.5

The samples were tested in triplicate and the experiment was repeated twice. The data are presented as mean ± SD.

**Table 2:** Inhibitory activities of CP, CP-IDL and T20 against T2635 or T20 resistant strains.

T2635-resistant strains	IC50 (nM) of		
	CP	CP-IDL	T20
WT	195.2 ± 12	10.75 ± 1.2	68.89 ± 3.4
N113E	296.6 ± 22	7.99 ± 3.5	148.06 ± 2.7
N126K	461.6 ± 44	46.43 ± 16	187.63 ± 3.8
K154Q	>500	9.79 ± 2.9	59.78 ± 15
Q66R, N126K	>500	301.5 ± 34	>500
T20-resistant strains			
WT	180.8 ± 16	8.67 ± 1.6	58.45 ± 4.6
N42T, N43K	>500	382.5 ± 82	>500

The samples were tested in triplicate and the experiment was repeated twice. The data are presented as mean ± SD.

**Table 3:** N36- and N43-L6-CP-IDL diffraction data and refinement statistics.

Crystallographic data	N36-L6-CP-IDL	N43-L6-CP-IDL
Resolution (Å)	42.9–2.30 (2.34–2.30)	33.9–2.30 (2.34–2.30)
Wavelength (Å)	1.5418	1.5418
Measured reflections	18327	19003
Average redundancy	3.4 (3.0)	1.6 (1.4)
Mean I/σ(I)	10.5 (2.8)	16.4 (1.8)
Completeness (%)	99.6 (94.7)	92.3 (81.9)
*R*_merge_^a^	0.087 (0.405)	0.037 (0.428)
Refinement statistics		
Space group	P2_1_	P1
Cell parameters		
a (Å)	42.45	39.1
b (Å)	114.47	39.1
c (Å)	42.94	90.6
α (°)	90	90.0
β (°)	91.8	90.0
γ (°)	90	120.0
Reflections in working set	15515	18000
Reflections in test set	1711	969
*R*_cryst_^b^	0.2194	0.2003
*R*_free_^c^	0.2629	0.2467
r.m.s.d. bonds (Å)	0.002	0.003
r.m.s.d. angles (°)	0.447	0.497
Average *B*-factor (Å^2^)	39.6	49.7
No. of waters	171	142

Values in parentheses indicate the corresponding statistics in the highest resolution shell.

^a^*R*_merge_ = (*I*_i_ − ‹*I*_i_›|)/*I*_i_|, where *I*_i_ is the integrated intensity of a given reflection.

^b^*R*_cryst_ = (||*F*_o_| − |*F*_c_||)/|*F*_o_|, where *F*_o_ and *F*_c_ denote observed and calculated structure factors, respectively.

^c^*R*_free_ is equivalent to *R*_cryst_, but calculated using randomly chosen 10% (N36-L6-CP-IDL) or 5% (N43-L6-CP-IDL) reflections as the test set, which were excluded from the refinement process.

## References

[b1] Chan D. C. & Kim P. S. HIV entry and its inhibition. Cell 93, 681–684, 10.1016/S0092-8674(00)81430-0 (1998). 9630213

[b2] Lu M., Blacklow S. C. & Kim P. S. A trimeric structural domain of the HIV-1 transmembrane glycoprotein. Nat Struct Biol 2, 1075–1082, 10.1038/nsb1295-1075 (1995). 8846219

[b3] Jiang S., Lin K., Strick N. & Neurath A. R. HIV-1 inhibition by a peptide. Nature 365, 113, 10.1038/365113a0 (1993). 8371754

[b4] Wild C. T., Shugars D. C., Greenwell T. K., McDanal C. B. & Matthews T. J. Peptides corresponding to a predictive alpha-helical domain of human immunodeficiency virus type 1 gp41 are potent inhibitors of virus infection. Proc Natl Acad Sci USA 91, 9770–9774, 10.1021/bi9606962 (1994). 7937889 PMC44898

[b5] Zhu X. . Improved Pharmacological and Structural Properties of HIV Fusion Inhibitor AP3 over Enfuvirtide: Highlighting Advantages of Artificial Peptide Strategy. Sci Rep 5, 13028, 10.1038/srep13028 (2015). 26286358 PMC4541410

[b6] Chan D. C., Fass D., Berger J. M. & Kim P. S. Core structure of gp41 from the HIV envelope glycoprotein. Cell 89, 263–273, 10.1016/S0092-8674(00)80205-6 (1997). 9108481

[b7] Tan K., Liu J., Wang J., Shen S. & Lu M. Atomic structure of a thermostable subdomain of HIV-1 gp41. Proc Natl Acad Sci USA 94, 12303–12308, 10.1073/pnas.94.23.12303 (1997). 9356444 PMC24915

[b8] Weissenhorn W., Dessen A., Harrison S. C., Skehel J. J. & Wiley D. C. Atomic structure of the ectodomain from HIV-1 gp41. Nature 387, 426–430, 10.1038/387426a0 (1997). 9163431

[b9] Mink M. . Impact of human immunodeficiency virus type 1 gp41 amino acid substitutions selected during enfuvirtide treatment on gp41 binding and antiviral potency of enfuvirtide *in vitro*. J Virol 79, 12447–12454, 10.1128/JVI.79.19.12447-12454.2005 (2005). 16160172 PMC1211558

[b10] Lu J. . Rapid emergence of enfuvirtide resistance in HIV-1-infected patients: results of a clonal analysis. J Acquir Immune Defic Syndr 43, 60–64, 10.1097/01.qai.0000234083.34161.55 (2006). 16885776

[b11] Sista P. R. . Characterization of determinants of genotypic and phenotypic resistance to enfuvirtide in baseline and on-treatment HIV-1 isolates. AIDS 18, 1787–1794, 10.1097/00002030-200409030-00007 (2004). 15316339

[b12] Yu X. . Mutations of Gln64 in the HIV-1 gp41 N-terminal heptad repeat render viruses resistant to peptide HIV fusion inhibitors targeting the gp41 pocket. J Virol 86, 589–593, 10.1128/JVI.05066-11 (2012). 22013063 PMC3255876

[b13] Naito T. . SC29EK, a peptide fusion inhibitor with enhanced alpha-helicity, inhibits replication of human immunodeficiency virus type 1 mutants resistant to enfuvirtide. Antimicrob Agents Chemother 53, 1013–1018, 10.1128/AAC.01211-08 (2009). 19114674 PMC2650564

[b14] Qi Z. . Rationally designed anti-HIV peptides containing multifunctional domains as molecule probes for studying the mechanisms of action of the first and second generation HIV fusion inhibitors. J Biol Chem 283, 30376–30384, 10.1074/jbc.M804672200 (2008). 18662985 PMC2573079

[b15] Chong H. . The M-T hook structure is critical for design of HIV-1 fusion inhibitors. J Biol Chem 287, 34558–34568, 10.1074/jbc.M112.390393 (2012). 22879603 PMC3464562

[b16] He Y. . Identification of a critical motif for the human immunodeficiency virus type 1 (HIV-1) gp41 core structure: implications for designing novel anti-HIV fusion inhibitors. J Virol 82, 6349–6358, 10.1128/JVI.00319-08 (2008). 18417584 PMC2447044

[b17] Ingallinella P. . Addition of a cholesterol group to an HIV-1 peptide fusion inhibitor dramatically increases its antiviral potency. Proc Natl Acad Sci USA 106, 5801–5806, 10.1073/pnas.0901007106 (2009). 19297617 PMC2667053

[b18] Stoddart C. A. . Albumin-conjugated C34 peptide HIV-1 fusion inhibitor: equipotent to C34 and T-20 *in vitro* with sustained activity in SCID-hu Thy/Liv mice. J Biol Chem 283, 34045–34052, 10.1074/jbc.M805536200 (2008). 18809675 PMC2590714

[b19] He Y. . Design and evaluation of sifuvirtide, a novel HIV-1 fusion inhibitor. J Biol Chem 283, 11126–11134, 10.1074/jbc.M800200200 (2008). 18303020

[b20] Chong H. . The M-T hook structure increases the potency of HIV-1 fusion inhibitor sifuvirtide and overcomes drug resistance. J Antimicrob Chemother 69, 2759–2769, 10.1093/jac/dku183 (2014). 24908047

[b21] Lu L. . Structure-based discovery of Middle East respiratory syndrome coronavirus fusion inhibitor. Nat Commun 5, 3067, 10.1038/ncomms4067 (2014). 24473083 PMC7091805

[b22] Lu L. . HIV-1 variants with a single-point mutation in the gp41 pocket region exhibiting different susceptibility to HIV fusion inhibitors with pocket- or membrane-binding domain. Biochim Biophys Acta 1818, 2950–2957, 10.1016/j.bbamem.2012.07.020 (2012). 22867851

[b23] Lalezari J. P. . Enfuvirtide, an HIV-1 fusion inhibitor, for drug-resistant HIV infection in North and South America. N Engl J Med 348, 2175–2185, 10.1056/NEJMoa035026 (2003). 12637625

[b24] Lazzarin A. . Efficacy of enfuvirtide in patients infected with drug-resistant HIV-1 in Europe and Australia. N Engl J Med 348, 2186–2195, 10.1056/NEJMoa035211 (2003). 12773645

[b25] Wei X. . Emergence of resistant human immunodeficiency virus type 1 in patients receiving fusion inhibitor (T-20) monotherapy. Antimicrob Agents Chemother 46, 1896–1905, 10.1128/AAC.46.6.1896-1905.2002 (2002). 12019106 PMC127242

[b26] Liu S. . Interaction between heptad repeat 1 and 2 regions in spike protein of SARS-associated coronavirus: implications for virus fusogenic mechanism and identification of fusion inhibitors. Lancet 363, 938–947, 10.1016/S0140-6736(04)15788-7 (2004). 15043961 PMC7140173

[b27] Poor T. A. . Probing the paramyxovirus fusion (F) protein-refolding event from pre- to postfusion by oxidative footprinting. Proc Natl Acad Sci USA 111, E2596–2605, 10.1073/pnas.1408983111 (2014). 24927585 PMC4078851

[b28] Tong P. . An engineered HIV-1 gp41 trimeric coiled coil with increased stability and anti-HIV-1 activity: implication for developing anti-HIV microbicides. J Antimicrob Chemother 68, 2533–2544, 10.1093/jac/dkt230 (2013). 23794600

[b29] Otwinowski Z. & Minor W. Processing of X-ray diffraction data collected in oscillation mode. Method Enzymol 276, 307–326, 10.1016/S0076-6879(97)76066-X (1997). 27754618

[b30] Mccoy A. J. . Phaser crystallographic software. J Appl Crystallogr 40, 658–674, 10.1107/S0021889807021206 (2007). 19461840 PMC2483472

[b31] Emsley P. & Cowtan K. Coot: model-building tools for molecular graphics. Acta Crystallogr D Biol Crystallogr 60, 2126–2132, 10.1107/S0907444904019158 (2004). 15572765

[b32] Adams P. D. . PHENIX: a comprehensive Python-based system for macromolecular structure solution. Acta Crystallogr D Biol Crystallogr 66, 213–221, 10.1107/S0907444909052925 (2010). 20124702 PMC2815670

[b33] Nelson M. T. . NAMD: A parallel, object oriented molecular dynamics program. Int J Supercomput Ap 10, 251–268, 10.1177/109434209601000401 (1996).

[b34] MacKerell A. D. . All-atom empirical potential for molecular modeling and dynamics studies of proteins. J Phys Chem B 102, 3586–3616, 10.1021/jp973084f (1998). 24889800

[b35] Humphrey W., Dalke A. & Schulten K. VMD: Visual molecular dynamics. J Mol Graph Model 14, 33–38, 10.1016/0263-7855(96)00018-5 (1996). 8744570

