# Supplementary material for: Rational improvement of gp41-targeting HIV-1 fusion inhibitors: an innovatively designed Ile-Asp-Leu tail with alternative conformations
Source: Sci Rep. 2016 Sep 26;6:31983. doi: 10.1038/srep31983 (PMC5036048; doi:10.1038/srep31983)
Supplement: Supplementary Information [file srep31983-s1.doc]

# Rational improvement of gp41-targeting HIV-1 fusion inhibitors: an innovatively designed Ile-Asp-Leu tail with alternative conformations

Yun Z^hu1^,†, Shan ^Su2^,†, Lili Q**^i^**^n1^,†, Qian Wa^n^g2, Lei S**^h^**i1, Zhenxuan ^M^a2, Shibo Jia^ng2,3^,*, Lu ^Lu^**^2^,** *, Sheng **^Ye1^**,*, Rongguang Zha**^ng1,4,*^**

**^1^**National Laboratory of Biomacromolecules, Institute of Biophysics, Chinese Academy of Sciences, Beijing, 100101, China.

^2^Key Laboratory of Medical Molecular Virology of Ministries of Education and Health, Shanghai Medical College and Shanghai Public Health Clinical Center, Fudan University, Shanghai, 200032, China.

^3^Lindsley F. Kimball Research Institute, New York Blood Center, New York, New York 10065, USA

^4^National Center for Protein Science Shanghai, Institute of Biochemistry and Cell Biology, Shanghai Institutes for Biological Sciences, Chinese Academy of Sciences, Shanghai, 201210, China.

†These authors contributed equally to the work.

*Correspondence should be addressed to: R.Z. (rzhang@sun5.ibp.ac.cn), S.Y. (yesheng@moon.ibp.ac.cn), L.L. (lul@fudan.edu.cn) or S.B.J (shibojiang@fudan.edu.cn).

**Running title:** HIV-1 fusion inhibitor with Ile-Asp-Leu tail

# Supplementary Information


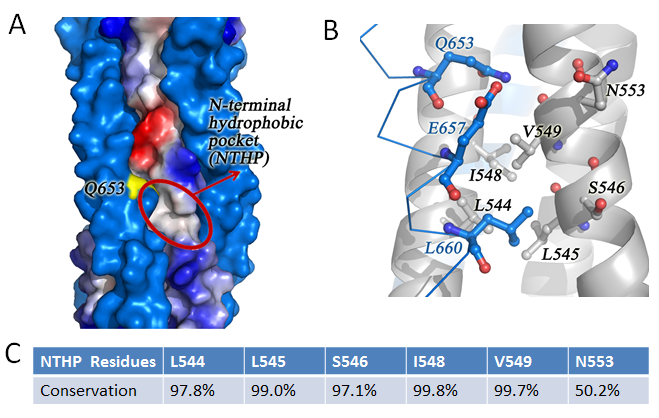


**Figure S1. Design HIV-1 fusion inhibitor based on the exposed N-terminal hydrophobic pocket (NTHP).** A, Crystal structure of gp41 fusion core (PDB: 2X7R). Three CHR are shown as surface in blue except for Q653 in yellow, and three NHR are shown as electrostatic surface. The NTHP is indicated by a red circle. B, important residues around NTHP are shown in sticks and labeled. C, the conservation of NTHP residues among 43622 different HIV-1 gp41 sequences.


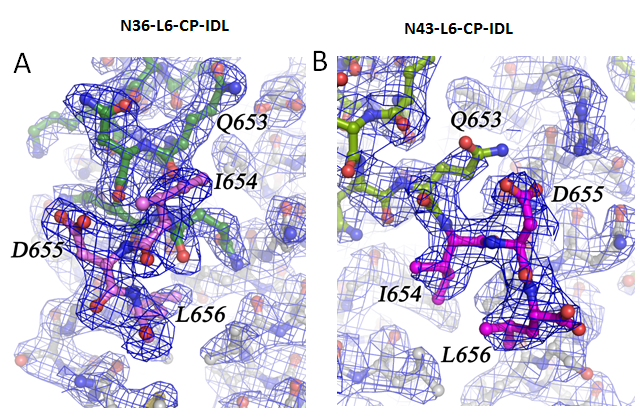


**Figure S2. IDL tail of the 6-HB structure formed by N36-L6-CP-IDL or N43-L6-CP-IDL displayed in stick model with the superimposed 2Fo-Fc electron density map.** The 2Fo-Fc electron density at 1.0 σ is shown in blue mesh.


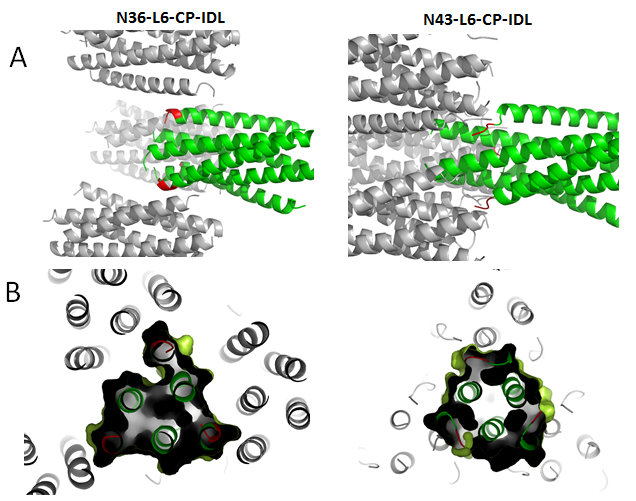


**Figure S3. IDL tails have direct interactions with adjacent 6HBs in both crystals of N36- and N43-L6-CP-IDL.** Crystal structures of N36- and N43-L6-CP-IDL are shown as cartoon representation in lateral view (A) and cross-section view (B). One 6HB is colored in green and red (IDL tail) with its surface showed, while other adjacent 6HBs are colored in grey.

**
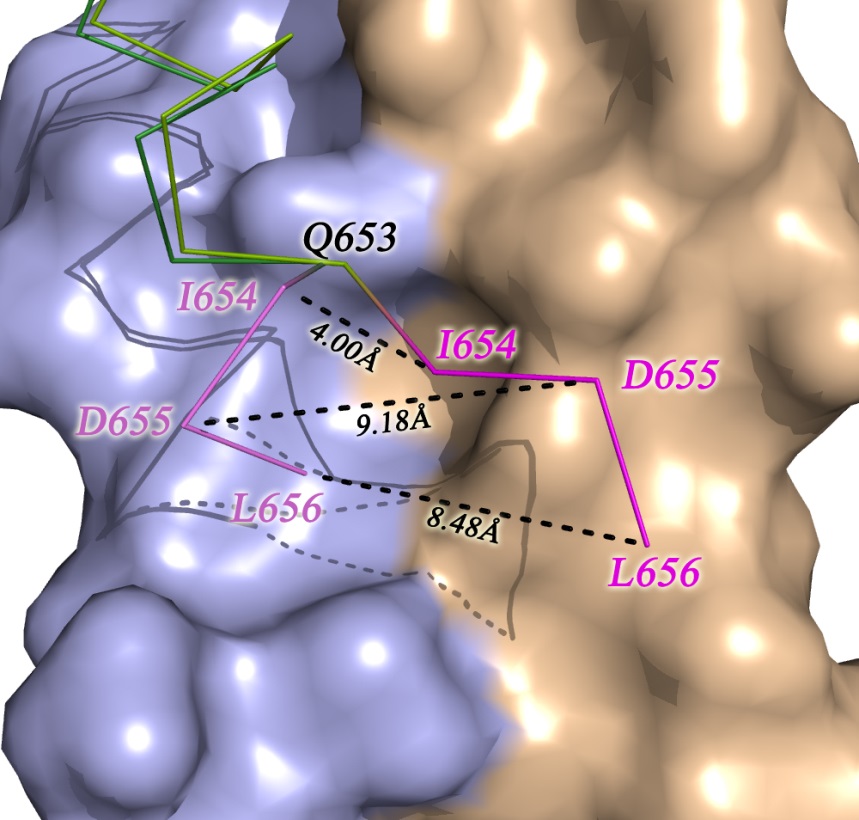
**

**Figure S4. Migration of IDL tail between two different conformations.** Crystal structure of N36-L6-CP-IDL and N43-L6-CP-IDL are shown as ribbon. Two NHR helices are shown as surface colored in wheat and light purple. The IDL tails are properly labeled and colored in violet or magentas in N36-L6-CP-IDL or N43-L6-CP-IDL, respectively.

# Tables

**Table S1. The r.m.s. deviation values between chains in crystal structure of N36-L6-CP-IDL and N43-L6-CP-IDL***

**N36-L6-CP-IDL**

| r.m.s. deviations (Å) | Chain A | Chain B | Chain C | Chain D | Chain E | Chain F |
| --- | --- | --- | --- | --- | --- | --- |
| Chain A | - | 0.48 | 0.82 | 0.38 | 0.50 | 0.66 |
| Chain B |  | - | 0.80 | 0.62 | 0.53 | 0.64 |
| Chain C |  |  | - | 0.88 | 0.59 | 0.70 |
| Chain D |  |  |  | - | 0.52 | 0.63 |
| Chain E |  |  |  |  | - | 0.48 |

**N43-L6-CP-IDL**

| r.m.s. deviations (Å) | Chain A | Chain B | Chain C | Chain D | Chain E | Chain F |
| --- | --- | --- | --- | --- | --- | --- |
| Chain A | - | 0.14 | 0.16 | 0.25 | 0.16 | 0.15 |
| Chain B |  | - | 0.14 | 0.16 | 0.12 | 0.13 |
| Chain C |  |  | - | 0.20 | 0.22 | 0.15 |
| Chain D |  |  |  | - | 0.30 | 0.17 |
| Chain E |  |  |  |  | - | 0.12 |

***** The structural superpositions were performed using the Secondary-Structure Matching (SSM)[^1^](#_ENREF_1) superposition function of program COOT[^2^](#_ENREF_2).

**Table S2. Average B factors of IDL tail in crystal structure of N36-L6-CP-IDL or N43-L6-CP-IDL**

**N36-L6-CP-IDL**

|  | **Chain A** | **Chain B** | **Chain C** | **Chain D** | **Chain E** | **Chain F** |
| --- | --- | --- | --- | --- | --- | --- |
| **Ile654** | 38.23 | 40.28 | 44.84 | 39.93 | n.a.* | 35.57 |
| **Asp655** | 42.65 | 53.10 | 62.87 | 43.49 | n.a. | 39.94 |
| **Leu656** | 43.46 | 50.27 | 67.06 | 48.83 | n.a. | 47.18 |

**N43-L6-CP-IDL**

|  | **Chain A** | **Chain B** | **Chain C** | **Chain D** | **Chain E** | **Chain F** |
| --- | --- | --- | --- | --- | --- | --- |
| **Ile654** | 57.90 | 58.86 | 56.13 | 56.28 | 56.05 | 52.82 |
| **Asp655** | 68.89 | 65.67 | 65.85 | 71.10 | 70.00 | 70.86 |
| **Leu656** | 77.53 | 71.90 | 79.22 | 80.28 | 76.32 | 78.75 |

***** The IDL tail in chain E of N36-L6-CP-IDL structure is missing in the final model.

# Movies

**Movie S1. Trajectory of isolated CP-IDL peptide with helical tail structure during the first 44 ns of a MD simulation run.**

# Supplementary References

1 Krissinel, E. & Henrick, K. Secondary-structure matching (SSM), a new tool for fast protein structure alignment in three dimensions. *Acta Crystallogr D Biol Crystallogr* **60**, 2256-2268, doi:S0907444904026460 [pii]

10.1107/S0907444904026460 (2004).

2 Emsley, P. & Cowtan, K. Coot: model-building tools for molecular graphics. *Acta Crystallogr D Biol Crystallogr* **60**, 2126-2132, doi:S0907444904019158 [pii]

10.1107/S0907444904019158 (2004).
